# Photonic integration based on a ferroelectric thin-film platform

**DOI:** 10.1038/s41598-019-52895-y

**Published:** 2019-11-12

**Authors:** Shunsuke Abe, Tomoki Joichi, Kouichiro Uekusa, Hideo Hara, Shin Masuda

**Affiliations:** Advantest Laboratories, Ltd., 48-1 Matsubara, Kamiayashi, Aobaku, Sendai, Miyagi 989-3124 Japan

**Keywords:** Integrated optics, Optoelectronic devices and components

## Abstract

Photonic-integrated circuits (PICs) using ferroelectric materials are expected to be used in many applications because of its unique optical properties such as large electro-optic coefficients. In this study, a novel PIC based on a ferroelectric thin-film platform was designed and fabricated, where high-speed optical modulator, spot-size converters (SSCs), and a variable optical attenuator (VOA) were successfully integrated. A ferroelectric lanthanum-modified lead zirconate titanate (PLZT) thin film was epitaxially-grown by using a modified sol-gel method, and it exhibits large electro-optic coefficients (>120 pm/V) and low propagation loss (1.1 dB/cm). The optical modulator, a Mach-Zehnder type, exhibited a half-wave voltage (V_π_) of 6.0 V (V_π_L = 4.5 Vcm_)_ and optical modulation up to 56 Gb/s. Also, the VOA (with attenuation range of more than 26 dB) was successfully integrated with the modulator. As a result, it is concluded that the developed ferroelectric platform can pave the way for photonic integration.

## Introduction

The increase in the capacity of data traffic requires high-speed and energy-efficient optical interconnects, and the demands for optical transceivers are increasing. Optical-interconnection devices are expected to be deployed in datacenters in high volume. Such devices today are mainly tested by using rack-and-stack solutions, and the testing has become a significant manufacturing bottleneck. Thus, in the same way as automated test equipment (ATE) is used in the CMOS semiconductor industry, ATE will be indispensable for reducing testing costs concerning optical devices. We have already proposed and demonstrated a concept for a high-volume optical-testing system^1–3^, which consists of high-speed electronics and optical components, such as light sources, optical modulators, photo-receivers, and variable optical attenuators (VOAs), and provides various electrical and optical test functions. To achieve high-volume optical testing using ATE, it is indispensable to integrate such bulky optical components in the testing system. In addition, to satisfy system requirements, the platform for the integration should be robust against high optical power.

Currently, optical modulators based on lithium niobate (LiNbO_3_ or LN hereafter) are widely utilized for optical communications and measurement systems. However, LN has an electro-optic (EO) coefficient^4^ of only 30 pm/V. Accordingly, LN modulators typically employ weakly guiding waveguides fabricated by titanium diffusion, so their resulting size is several centimeters in length. Also, their optical performance is sometimes deteriorated by a photorefractive effect during input of high-power optical signals.

Recently, EO polymers have attracted attention because of their large EO coefficient and ultrafast response to an applied electric field^5,6^. Modulation bandwidth of EO-polymer modulators is more than 100 GHz at low driving voltage, resulting in merits such as eliminating the need for a high-power electrical RF amplifier^7^. Although EO-polymer modulators achieve excellent performance, optical loss of their polymer waveguides is not low enough to allow them to be integrated with other components. Also, in regard to high-optical-power and long-term operation, reliability of the EO chromophores could be an issue.

In the meantime, over the last few decades, silicon photonic devices have received much attention^8–10^ because they can be fabricated on silicon-on-insulator (SOI) substrates by using a CMOS-compatible process. Accordingly, it is expected that not only photonic devices but also electrical ones can be integrated on a single chip. However, silicon modulators based on carrier plasma effects have a theoretical bandwidth limitation of 40 GHz^11^, and their optical signal linearity deteriorates above that limit^12^. To overcome this bandwidth limitation, silicon-organic hybrid (SOH) modulators, which utilize EO polymers with Pockels effect on a silicon platform, were demostrated^13,14^. Although SOH modulators can be expected to achieve both high-speed optical modulation and photonic integration because of their large EO coefficient, their fabrication process needs to be further improved to reduce optical propagation loss of the highly confined slot waveguides.

Ferroelectric materials are also one of the candidates for photonic integration because of their large-EO coefficient^15,16^ and robustness. Mach-Zehnder (MZ) optical modulators using ferroelectric materials have long been studied^17,18^, and ferroelectric modulators using a silicon or ultra-low-loss silicon-nitride (SiN) platform have been studied recently. For example, a plasmonic BaTiO_3_ modulator on silicon^19^ and a PZT ring modulator on SiN^20^ demonstrated the potential of ferroelectric materials for modulators and photonic integration. Although PLZT bulk ceramic has a large EO coefficient (>700 pm/V), previously reported PLZT thin films had a small EO coefficient. In our previous study, we revealed the difference between the crystal phase of bulk ceramics and thin films synthesized by a conventional sol-gel process at the same composition ratio. To synthesize epitaxially-grown PLZT thin films with high crystallinity and large EO properties, we have developed the “modified sol-gel process”^21^. With this process, we have successfully controlled the crystal phase of the PLZT so as to enhance the EO coefficient of the films. Using these ferroelectric oxide thin films, we have developed a photonic integrated circuit (PIC)^22,23^, where waveguides with a loss of 1.1 dB/cm, VOAs, and a high-speed optical modulator operating up to 50 Gb/s were integrated.

In the present study, we describe how we improve the device performances such as the bandwidth, half-wavelength voltage, and optical coupling losses of the PIC chip. As a result, we have successfully integrated newly-developed spot-size convertor (SSC), optimally-designed PLZT modulator and VOA on a PLZT platform. Additionally, to provide various optical test functions in a single package for ATE, an optical transmitter module, which consists of laser diode (LD), photo-detectors (PDs), and optical fiber connected with the PIC chip, was realized.

## Results

### Design of PIC using PLZT on sapphire

**Figure 1:**
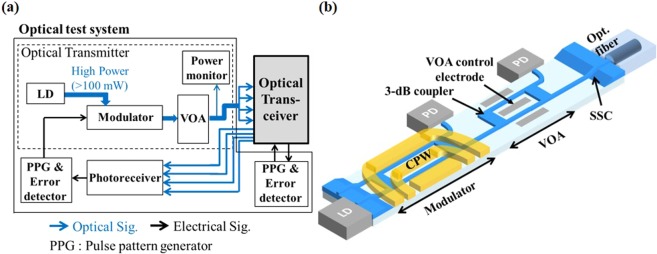
Schematic of (**a**) the test system and (**b**) the PLZT photonic integrated circuit.

The configuration of an optical test system for high-volume testing is shown schematically in Fig. 1(a). The system consists of optical signal transmitters, receivers and high-speed electronics for testing high-speed optical interfaces such as optical transceivers. As shown in the figure, various optical components, such as light sources, VOAs, optical switches, and photoreceivers, are embedded in the system. Here, an output optical test signal from the optical modulator has to be divided into four channels or more to established simultaneous testing. So, we cannot utilize the LN modulator in the optical test system due to its photorefractive effect, which sometime deteriorates the optical performances of the LN modulator under the condition of high power operation. As shown in Fig. 1(b), therefore, the PLZT PIC was designed as an optical transmitter for the system. A high-speed modulator, a VOA, and SSCs are monolithically integrated on the PLZT film deposited on a sapphire substrate. Furthermore, a proprietary optical-assembly technique was used to directly couple the monitor PDs, a LD, and an optical fiber to the PIC, which provided a low-optical-loss implementation of the transmitter module. Input light generated by the LD is fed into the PLZT PIC and converted into non-return-to-zero (NRZ) high-speed optical signals by the optical modulator. For sensitivity tests of optical transceivers, optical power can be varied by the VOA, and the power level of each signal can be monitored by the monitor PDs.

### Design of optical waveguide, SSC, and 3-dB coupler

**Figure 2:**
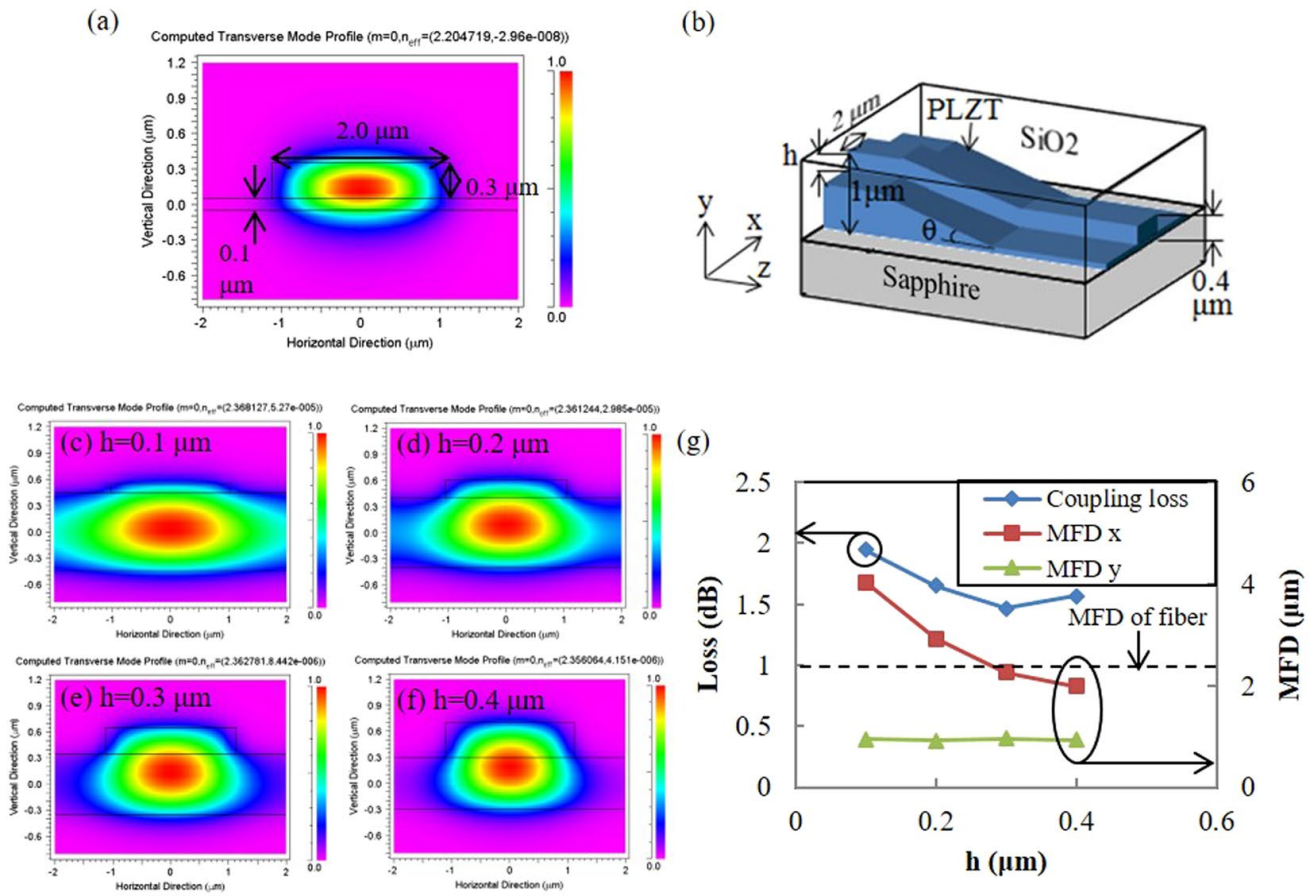
Design of the optical waveguide and SSC using FDTD: (**a**) optical field distribution of PLZT waveguide, (**b**) Simulation model of SSC connected with PLZT waveguide, and its optical field distributions of SSC at ridge height (*h*) from 0.1 to 0.4 μm (c–f). (g) MFDs and coupling losses of SSCs of each *h*. For each calculation, TE-mode Gaussian beam is input, computational windows are x = −2 μm to 2 μm, y = −0.8 μm to 1.2 μm, grid size of which are all 0.05 μm. Refractive indices of PLZT, SiO_2_ and sapphire are 2.42, 1.44 and 1.76, respectively.

To design the structures of the PLZT waveguides and SSCs, a finite-difference time-domain (FDTD) method was utilized. A ridge-type single-mode waveguide was designed for transverse electric (TE) mode at a wavelength of 1310 nm. The results of applying FDTD to the PLZT waveguide, with width and height of 2.0 and 0.3 μm, respectively, are shown in Fig. 2(a). From the FDTD results, single-mode propagation in this structure was confirmed, and the horizontal and vertical mode field diameters (MFDs) of the waveguide were 1.78 and 0.48 μm, respectively. Here, MFD is defined by the point at which the optical power reduces to 1/e^2^ of the maximum power. To compensate for the mismatch between MFDs of the waveguides and lensed fibers (~2.3 μm), the SSC shown in Fig. 2(b) was designed. The SSC consists of a vertically up-tapered slope with angle *θ* and a ridge with height *h*. Firstly, dependence of SSC propagation loss on *θ* was simulated. With increasing *θ*, propagation loss of the SSC increases due to light leakage from the tapered waveguide, and the loss is negligible when *θ* is less than 9 degrees. Ridge height (*h*) is optimized to minimize the optical-coupling loss as shown in Fig. 2(c–f). While vertical MFD (MFD_y_) stays almost constant with increasing *h*, horizontal MFD (MFD_x_) decreases because the propagation light in the SSC is almost confined in the waveguide. As summarized in Fig. 2(g), MFDs and coupling loss of the SSC, were calculated from the following equation, which is given by overlap of fields of the SSC and lensed fiber as

1 $$\eta ={|\int {\varphi }_{1}\cdot {\varphi }_{2}dS|}^{2},$$

where *ϕ*_1_ and *ϕ*_2_ denote optical intensity distributions of the MFDs of the SSC and lensed fiber. Here, the MFD of the lensed fiber is 2.3 μm. Optical coupling loss between the SSC and lensed fiber has a minimum value at *h* = 0.3 μm, because MFD_x_s coincided with each other. Compared with the optical-coupling loss of the waveguide without the SSC (3.41 dB), that of the waveguide with the SSC is reduced to 1.47 dB, which is 3.88 dB lower on both facets. The loss could be further reduced if thickness of the PLZT film is increased so that MFD_y_ of the SSC can be matched with that of the fiber.

Multi-mode interference (MMI) couplers^24^ were utilized for splitting and coupling signal lights. A beam-propagation method (BPM) was used to design the MMI coupler. From the BPM calculation, optimum values of width and length were determined as 7.5 μm and 240 μm with excess loss of less than 0.2 dB (see Fig. S5 in Supplementary Information).

### Design of PLZT modulator

**Figure 3:**
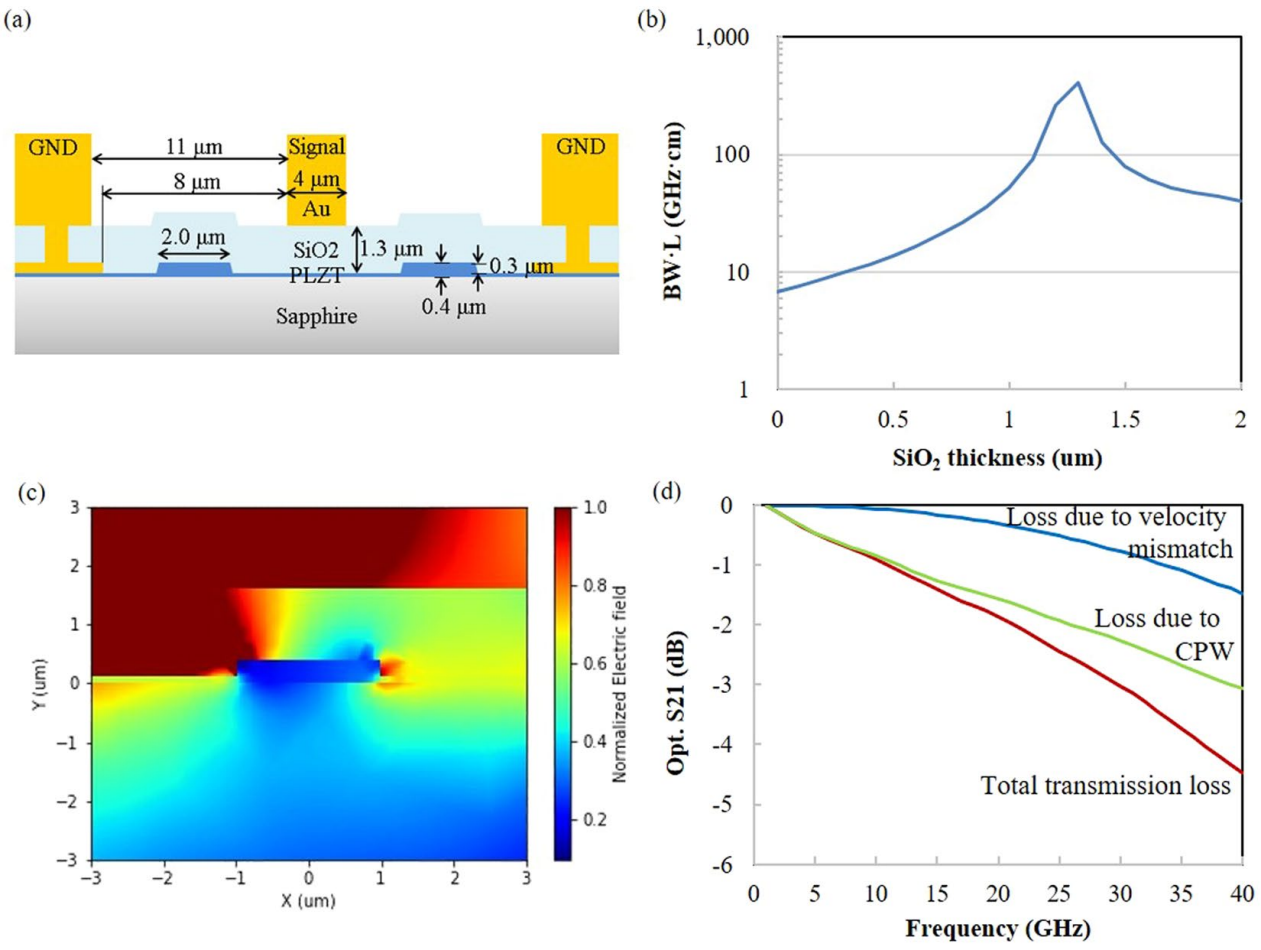
(**a**) Cross-sectional image of the PLZT modulator. (**b**) Bandwidth limitation of the PLZT modulator induced by velocity mismatch between optical wave and microwave, which can be adjusted by SiO_2_ thickness. (**c**) Electrical field distribution of PLZT modulator. Electrical signals (1 GHz to 40 GHz) were input to signal electrode, and the grid size of x and y are both 0.02 μm. (**d**) Simulated optical responses of the PLZT modulator (S_21_).

A high-speed Mach-Zehnder-interferometer-type PLZT thin-film modulator was designed, which utilizes Pockels effect. To design the PLZT modulator, optical-modulation bandwidth is limited by the large permittivity (ε_r_) of the PLZT film (ε_r_ = 700) and velocity mismatch between optical waves and microwaves. To reduce the effective permittivity, PLZT was sandwiched between low-ε_r_ dielectrics as shown in Fig. 3(a). A sapphire (ε_r_ = 10) substrate and a SiO_2_ (ε_r_ = 4) buffer layer were utilized as low-ε_r_ dielectrics, and effective dielectric constant of the PLZT waveguide was reduced to 7.7 (according to calculation).

To achieve velocity matching of the optical wave and microwave for high-speed operation, thickness of the SiO_2_ buffer layer was optimized. The -3-dB bandwidth induced by the velocity mismatch (*BW*_*m*_) is expressed by^25^

2 $$B{W}_{m}\approx \frac{1.90c}{\pi }\frac{1}{|{n}_{o}-{n}_{m}|L},$$

where *L* is the length of the electrode, *n*_*o*_ and *n*_*m*_ respectively denote effective refractive indexes of the optical wave and microwave. According to Eq. (2), by changing the thickness of SiO_2_, the bandwidth limitation caused by velocity mismatch *n*_*m*_ can be controlled. Dependence of *BW*_*m*_ on SiO_2_ thickness is shown in Fig. 3(b), which indicates that optimal thickness is 1.3 μm. In addition, a 50-Ω coplanar waveguide (CPW), with width and gap of 4 μm and 8 μm was designed. The -3-dB optical bandwidth due to conductor loss and skin effect of the CPW (*BW*_*loss*_) is given by

3 $$B{W}_{loss}={(\frac{3.18}{{\alpha }_{0}L})}^{2},$$

where *α*_0_ is the electrode attenuation constant. The -3-dB optical bandwidth of the modulator is roughly estimated by the sum of the bandwidths given by Eqs (2) and (3).

*V*_*π*_ of the modulator is given by^26^

4 $${V}_{\pi }=\frac{\lambda d}{2{n}^{3}rL\varGamma },\,\Gamma =\frac{\iint {e}^{2}(x,y)E(x,y)dxdy}{E\iint {e}^{2}(x,y)dxdy},$$

where *r* is the EO coefficient of the PLZT film, *λ* is the wavelength of light, *d* is the electrode separation, *n* is optical refractive index of the PLZT film, and *x* and *y* denote the axes in the plane of the cross section of the waveguide, respectively. *Γ* represents a normalized overlap of electrical field (*E*) of microwaves and the square of optical field (*e*). Field distribution of PLZT modulator calculated by FEM is shown in Fig. 3(c). The results of FDTD (Fig. 2(a)) and FEM are used to calculate *Γ* from Eq. (4), and *V*_*π*_*L* = 3.9 Vcm can be derived. According to Eqs (2–4), the length of the CPW determines the trade-off between *V*_*π*_ and optical bandwidth of the modulator. Optical bandwidth and *V*_*π*_ can thus be roughly calculated so as to fit the system specification (i.e., bandwidth of the modulator >24 GHz). As *L* was determined to be 7.5 mm (*V*_*π*_ = 5.2 V). Optical-frequency response of this structure, which can be calculated as the sum of the transmission losses due to velocity mismatch and the CPW, is shown in Fig. 3(d). The optical loss due to the CPW was calculated from the result of electrical-frequency response simulated by FEM. The -3-dB optical bandwidth of the modulator and electrical -6-dB bandwidth of the CPW were calculated to be 30 GHz and 35.9 GHz, respectively. As can be seen in Fig. 3(d), transmission loss due to the velocity mismatch is relatively small because of the velocity-matched design, and optical-frequency response depends on electrical transmission loss of the CPW.

### Fabrication of PIC

Epitaxially-grown PLZT thin films were synthesized using the modified sol-gel method^21,22^. The main feature of this synthesis is that the film is crystallized under a suitable oxygen gas pressure, whereas the conventional crystallization process is usually performed by flowing oxygen gas under atmospheric pressure. This process enable us to control crystal phase of the PLZT film to enhance the electrooptic coefficients, and the epitaxially-grown PLZT(8/65/35) film with rhombohedral-phase can be synthesized without pyrochlores, lead, or oxygen deficiencies. The root-mean-square (RMS) surface roughness of the film was measured by an atomic force microscopy (AFM) as 0.23 nm. The EO coefficient was estimated to be 120 pm/V, which is almost four times larger than that of LiNbO_3_ crystals.

**Figure 4:**
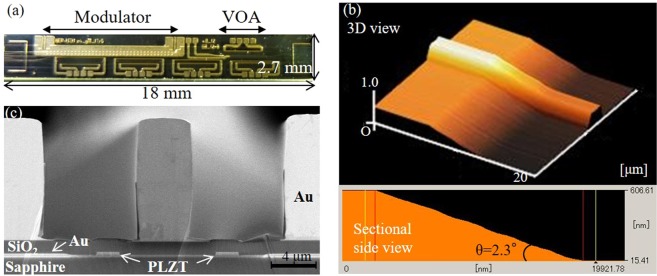
Fabricated PLZT PIC. (**a**) Microphotograph of the fabricated PLZT PIC chip (top view). (**b**) AFM image of SSC, where the angle of the up-taper slope is 2.3 deg. (**c**) Cross-sectional image of the PLZT modulator section.

A microphotograph of the PLZT PIC chip is shown in Fig. 4(a). The ridge waveguide and SSC structure were formed by dry etching. The up-tapered slope of the SSC was shaped by step-and-exposure lithography and dry etching. An AFM image of the SSC is shown in Fig. 4(b). The inclination angle of the SSC was less than 9° (*θ* = 2.3°), and the thickness of PLZT was reduced by almost 0.6 μm as designed. Roughness average (RA) of the slope was 4.2 nm, which is smooth enough for the wavelength of light (1310 nm). A cross-sectional scanning-electron-microscopy (SEM) image of the fabricated optical modulator is shown in Fig. 4(c). The PLZT waveguide was sandwiched between sapphire and a 1.3-μm-thick SiO_2_ film. Lower electrodes were formed for poling of the PLZT film, and an upper electrode (CPW) was fabricated by gold plating. Thicknesses of lower and upper electrodes were 0.3 and 12 μm, respectively. Here, the single-mode waveguide was aligned at 45 degrees with respect to the <001> crystallographic direction of the PLZT to obtain the maximum EO coefficient for the TE mode with a coplanar electrode structure shown in Fig. 3(a), since the direction of the spontaneous polarization of the PLZT is coincident with the incident light.

**Figure 5:**
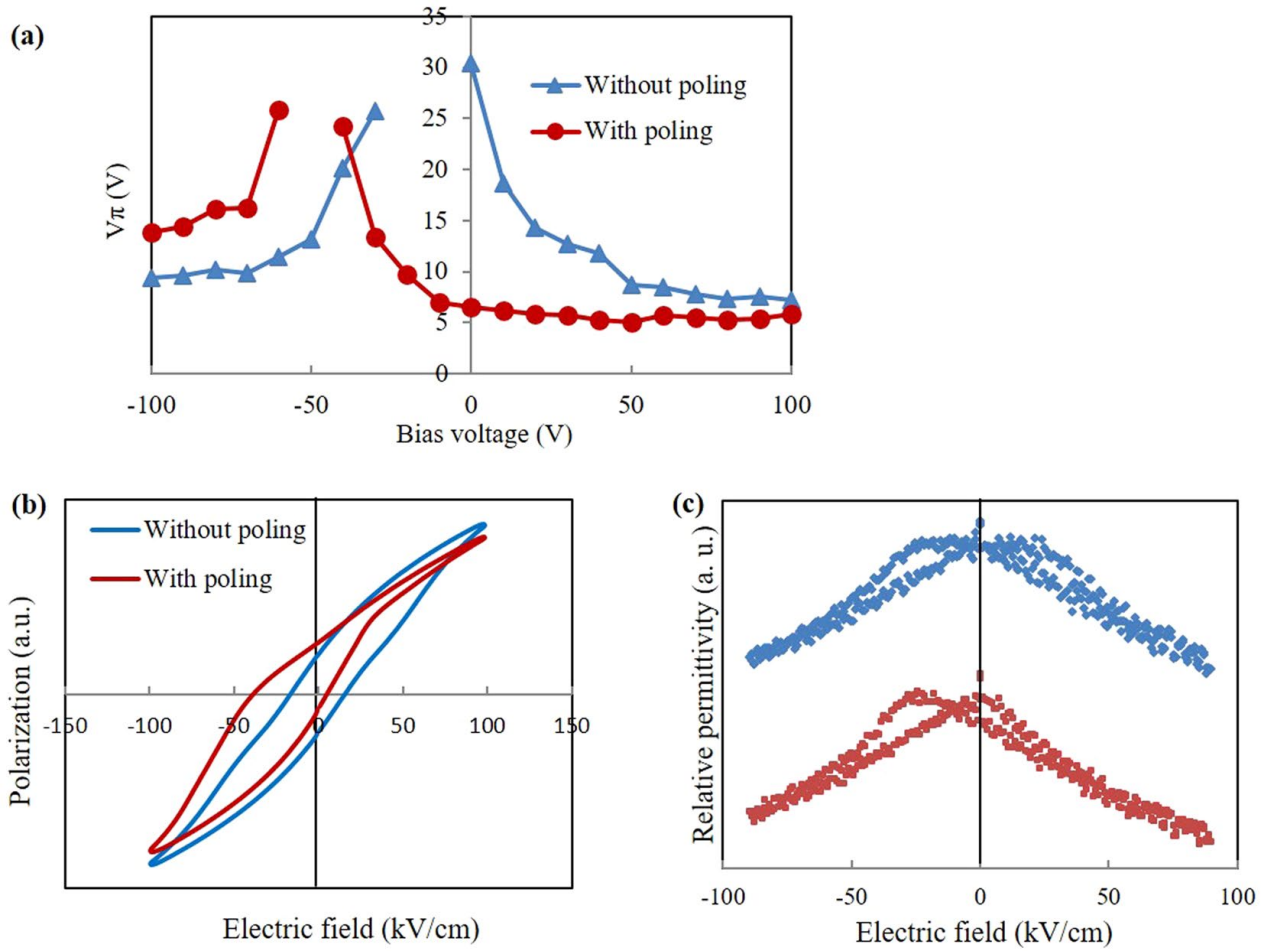
(**a**) Bias-voltage dependence of *V*_*π*_. (**b**) P-E hysteresis loop of PLZT formed with and without poling process. (**c**) Relationships between relative permittivity and applied electric field (butterfly curves) of the modulator with and without poling.

To realize bias-voltage-free operation of the PIC, ferroelectric polarization imprint, which is realized by the poling process and reduces the coercive electric field of ferroelectrics, was introduced^27,28^. Dependence of *V*_*π*_ on bias voltage, in the cases with and without application of poling, is shown in Fig. 5(a). The bias voltage was applied to the GND electrodes on the left and right sides of the signal electrode (see Fig. 3(a), where the poling direction is defined as positive. In the case without poling, *V*_*π*_ is about 30 V when bias voltage is 0 V. Hence, it is necessary to apply bias voltages to the PLZT film to attain low-V_π_ operation. On the other hand, after poling, it appears that the *V*_*π*_-bias-voltage curve shifted in the negative direction. *V*_*π*_ is high when bias voltage is around −60 to −40 V, and it decreases as bias voltage increases in either the positive or negative direction. *V*_*π*_ is drastically reduced to 6.0 V even when bias voltage is 0 V. To examine the imprint behavior of the PLZT film, P-E hysteresis loops and permittivity butterfly curves in the cases with and without poling were confirmed. P-E hysteresis loops of the optical modulator are shown in Fig. 5(b). The sample without poling shows a typical symmetric hysteresis curve of ferroelectrics, while the sample with poling shows an asymmetric curve. The positive (*E*_*C+*_) and negative (*E*_*C-*_) coercive electric fields, where polarization *P* = 0, are respectively 15.1 and −13.7 kV/cm without poling and respectively 4.1 and −37.3 kV/cm with poling. The shift can also be seen in relationships between relative permittivity and applied electric field (permittivity butterfly curves) as shown in Fig. 5(c). The shifts in the hysteresis loop and the butterfly curve can be explained by a ferroelectric polarization-pinned layer formed on the interface between the ferroelectrics and electrodes^27,28^. Since the pinned layer reduces *E*_*C*+_ of the PLZT thin film by poling, bias-voltage-free operation of the PLZT optical modulator can be obtained.

### Design and evaluation of VOA

Mach-Zehnder-interferometer-type VOA was designed, which is also operated based on Pockels effect of the PLZT thin film. To realize high-speed response and small-size device, signal and ground electrodes with the gap of 8 μm were directly deposited on the PLZT film, which enables to apply the electric field to the PLZT waveguide 7.5 times higher than the modulator mentioned previously. Considering the control of attenuation and its resolution of the VOA, the interaction length was determined to be 2 mm. Consequently, *V*_*π*_ and attenuation range of the fabricated VOA were 2.4 V (*V*_*π*_*L* = 0.58 Vcm) and 26 dB, respectively, whose settling time was measured to be 40 nanoseconds. By operating VOA and modulator in the PIC, we have confirmed that the power levels of high-speed signals are controlled arbitrarily, which realizes sensitivity tests of photoreceivers up to 56 Gb/s.

### Evaluation of fabricated PIC

Optical insertion loss (IL) of the PLZT PIC was measured as 8.0 dB (including optical-coupling loss due to the optical fibers), while that of the PIC without the SSC was 11 dB. This result indicates that the PLZT-waveguide-integrated SSC reduces optical coupling loss by 3 dB. The difference between ILs of the simulation (in Fig. 2(g)) and the experiment is believed to be caused by the surface roughness of the up-tapered slope or the sidewall of the PLZT waveguides.

**Figure 6:**
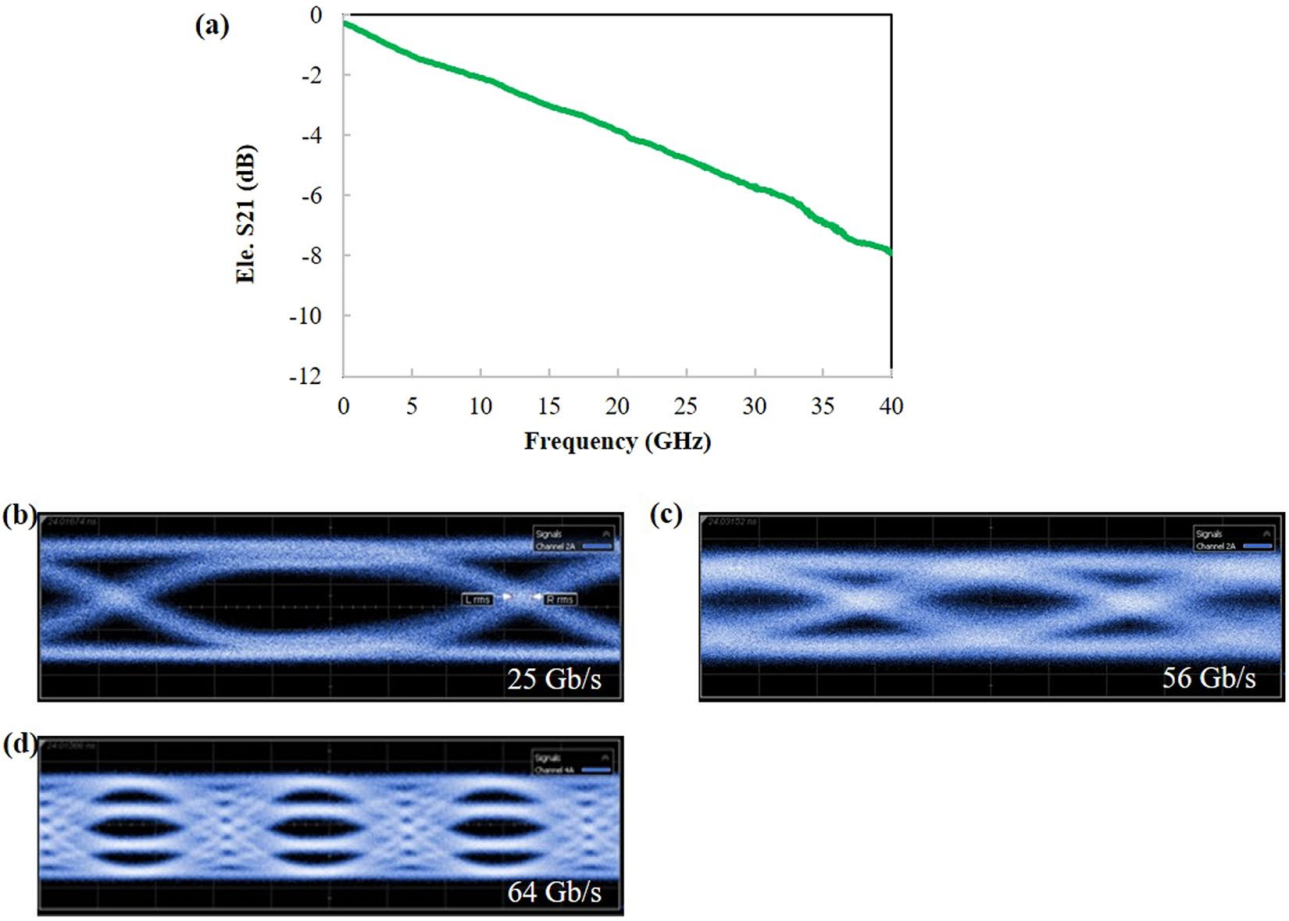
(**a**) Electrical frequency response (*S*_21_) of the PLZT modulator and optical eye diagrams operated at (**b**) 25 Gb/s and (**c**) 56 Gb/s. (**d**) 64 Gb/s 4-level pulse amplitude modulation (PAM-4). Outer extinction ratio was 6 dB.

Frequency response (S_21_) of the CPW is shown in Fig. 6(a), which indicates −6-dB bandwidth is 31.9 GHz. As can be seen in the figure and from the previously described FEM calculation, -3-dB optical bandwidth is estimated to be 28.4 GHz. Also, high-speed operation of the PLZT PIC was examined by using a pulse-pattern generator. Optical eye diagrams of the PLZT modulator operating at 25 and 56 Gb/s are shown in Fig. 6(b,c), respectively. As can be seen in both figures, a clear eye opening was observed up to 56 Gb/s, where the rise and fall times for 56-Gb/s operation were measured as 11.3 and 10.0 psec., respectively. Extinction ratios (ER) of these eye diagrams were measured to be 6.3 dB and 4.5 dB. Figure 6(d) represents optical four-level pulse amplitude modulation (PAM-4) at 64 Gb/s (32 Gbaud). Open PAM-4 eye was observed, and its extinction ratio was measured to be 6 dB.

## Discussion

A novel photonic-integrated circuit was successfully developed by using an epitaxially-grown PLZT thin film. A high-speed optical modulator, SSCs, and a VOA were designed and fabricated. Poling was utilized to control polarization of PLZT, and V_π_ was drastically reduced without applying bias voltage. As a result, the optical modulator (operating at up to 56 Gb/s) and the VOA (with attenuation ratio of more than 26 dB and operating within 40-nanosecond response time) were integrated on a single chip. The PLZT PIC can provide an optical transmitter for ATE in a small package and can reduce the testing time. The PLZT platform is also advantageous in terms of its unique material properties, namely, thermal stability and robustness against high optical power. Large-EO coefficient (120 pm/V) with relatively low propagation loss (1.1 dB/cm) is one of the advantages of PLZT compared with other EO materials such as EO polymer. Therefore, the PIC platform developed in this study is promising for a high-optical-power system such as ATE.

## Methods

Epitaxially grown PLZT thin films were synthesized by using a modified sol-gel method. In this synthesis, commercially available precursor solutions (Mitsubishi Materials Co.), consisting of 15-wt% precursor with La:Ti:Zr ratio of 8:65:35 and 20-wt% excess lead, were utilized. The PLZT solution was spin-coated on an *r*-cut sapphire substrate and pyrolyzed at 300 °C for 3 min in air. The wafer was annealed in oxygen at around 625 °C for 3 min by rapid thermal annealing (RTA). The synthesis of the PLZT films is described in detail in a previous paper^21^.

PLZT PICs were fabricated by photolithography using a g-line stepper. To fabricate the shape of the SSC, step-and-exposure lithography was used. The fabrication procedure is explained as follows. First, the photoresist layer is repeatedly exposed to weak light while the photo-mask is moved in several-micron steps. Since exposure time of the photo-resist layer is gradually varied, a stepwise shape can be formed after the developing process. Then, a smooth up-tapered slope photo-resist layer can be formed after the baking process. The angle of the up-tapered slope can be controlled by the distance of the movement step of the photo mask. The photo-resist pattern is transferred by dry etching, i.e., electron-cyclotron-resonance etching (ECR) with C_4_F_8_- and argon-based chemistry.

Chromium/gold/chromium lower electrodes were deposited by RF sputtering using argon gas and were formed by a lift-off process, where chromium was used for the adhesion layer to PLZT and SiO_2_. The upper CPW electrode was formed by gold plating and milling. SiO_2_ was deposited by RF-plasma sputtering using argon and oxygen gas at room temperature. To reduce bias voltage, the PLZT film was poled by applying a coercive electric field above the Curie temperature (>110 °C) via the lower electrode.

For measuring eye-diagrams, a pulse-pattern generator (N4975A, Keysight) with 2^15^−1 non-return-to-zero electrical signals and a driver amplifier (OA4MVM3, Centellax) were used to drive the PLZT modulator. Also, PAM-4 electrical signals were generated by an arbitrary waveform generator (M8194, Keysight Technologies) and a driver amplifier (in-house developed gallium nitride amplifier). Optical signals were measured by using an optical sampling oscilloscope with a 45-GHz optical bandwidth (86116C, Keysight Technologies).

## Supplementary information

supplementary information
