# Supplementary material for: Photonic integration based on a ferroelectric thin-film platform
Source: Sci Rep. 2019 Nov 12;9:16548. doi: 10.1038/s41598-019-52895-y (PMC6851366; doi:10.1038/s41598-019-52895-y)
Supplement: Supplementary file 1 — supplementary information [file 41598_2019_52895_MOESM1_ESM.pdf]

Supplementary information

Photonic integration based on a ferroelectric thin-film platform

Shunsuke Abe[1], Tomoki Joichi[1], Kouichiro Uekusa[1], Hideo Hara[1] and Shin Masuda[1*]

Epitaxially-grown PLZT thin films

Epitaxially-grown PLZT thin films were synthesized using the modified sol-gel method. The RMS surface roughness of the film was measured by an atomic force microscopy (AFM) as 0.23 nm (Fig. S1). To check the crystallinity of PLZT thin film, X-ray diffraction (XRD) measurement was conducted. As can be seen in Figs. S2a and S2b, PLZT (110) thin film was epitaxially-grown on a *r*-cut sapphire substrate. The absorption and EO coefficient of the film were measured to be 1.0 dB/cm and 120 pm/V, respectively.

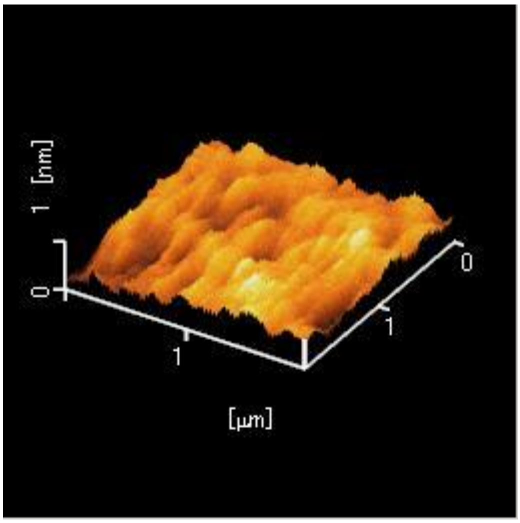

Figure S1 | Atomic force microscopic image of epitaxially-grown PLZT thin film on a sapphire substrate. The RMS surface roughness of the film was measured as 0.23 nm.

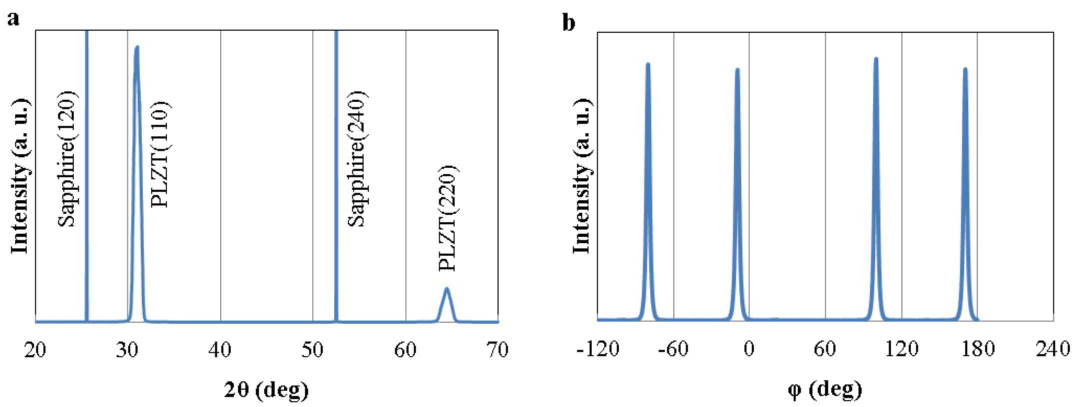

Figure S2 | X-ray diffraction Patterns of the PLZT thin film. a, The results indicate PLZT thin film is expitaxially-grown on the *r*-cut sapphire substrate. **a,** 2θ-θ measurement. A single (110) PLZT diffraction profile was observed except for the peaks attributed to the sapphire substrate, which indicates the film is highly oriented to (110) direction. **b,** ϕ–scan measurement. The peaks show the in-plane orientation and high crystallinity of the film.

Fabrication of SSC using step-and-exposure lithography

Up-tapered shapes were realized using step-and-exposure lithography and dry etching. The procedure is described in Figure S3. The main feature of this process is the inclination angle θ can be controlled by stepper or etching condition. Given photoresist thickness h_{PR}, threshold-exposure power P_{th} (a power that makes a photoresist start to develop) and resolution-exposure power P_{res} (a power that makes a photoresist completely develop), θ is roughly estimated as

$$\theta = \tan^{-1}\left(\frac{\Delta P}{\Delta l}\cdot\frac{xh_{PR}}{P_{res}-P_{th}}\right), \quad \text{(S1)}$$

where x is selectivity of etching, Δl and ΔP are a length of a moving step and a exposure power, respectively. x is a ratio of etching rates of PLZT and photoresist, which depends on etching condition. Δl and ΔP can be controlled by stepper. Figure S4 represents the optical micrograph of the fabrication process.

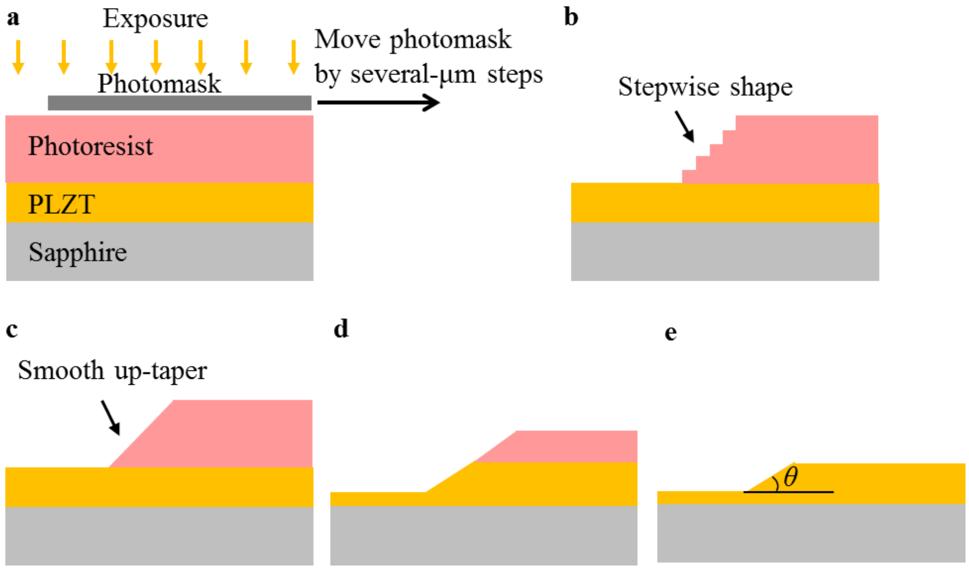

Figure S3 | Fabrication process of up-tapered slope using step-and-exposure lithography. a, Photoresist-coated epitaxiall-grown PLZT thin film. The photoresist layer is repeatedly exposed to weak light while the photo-mask is moved in several-micron steps. **b,** Since exposure time of the photo-resist layer is gradually varied, a stepwise shape can be formed after the developing process. **c,** A smooth

up-tapered slope photo-resist layer after the baking process. **d,e,** After dry etching (**d**) and photoresist removal (**e**).

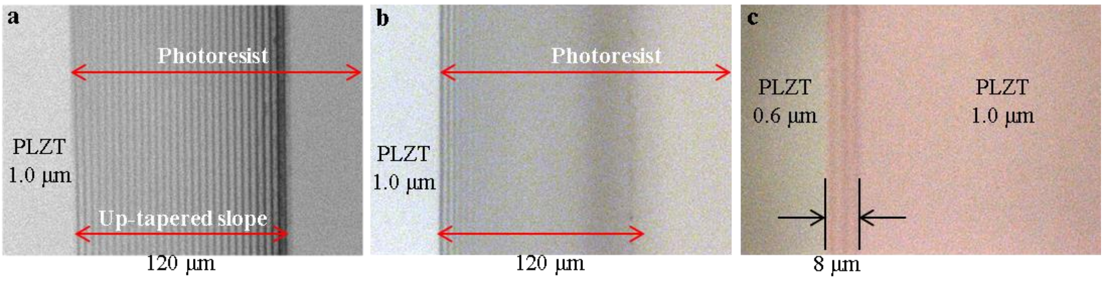

Figure S4 | Optical micrograph of fabrication process of up-tapered slope. a, A stepwise shape of the photoresist taken after light exposure and developing. **b,** Smooth up-tapered slope of photoresist realized by baking. **c,** Up-tapered slope of PLZT. PLZT was etched to 0.6 µm and the photoresist was removed. As can be seen in Fig. 4b, smooth up-tapered shape was derived. Length of the slope was 8 µm.

Design of MMI coupler

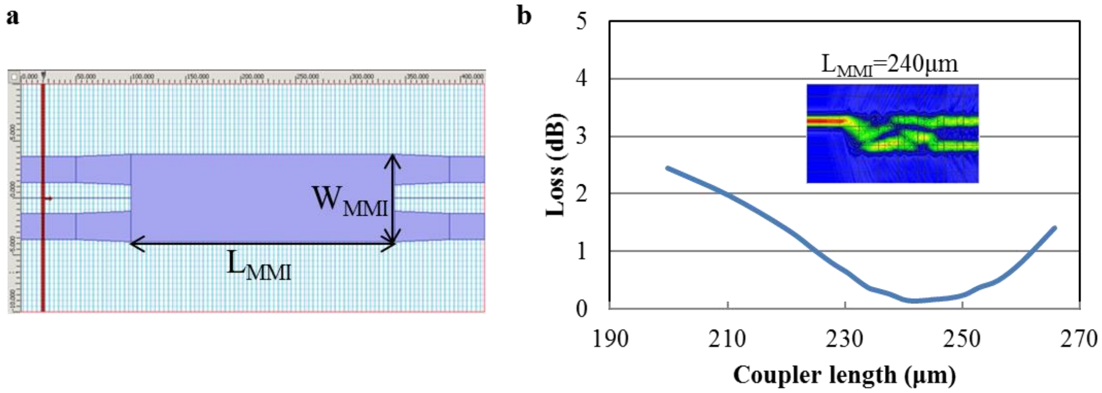

Figure S5 | Design of MMI PLZT coupler. Beam propagation method (BPM) was utilized. **a,** Simulation model. Cross-section of the model is the same with Fig. 2a. **b,** Dependence of excess loss on L_{MMI} at W_{MMI}=7.5 µm. From the BPM calculation, optimum values for W_{MMI} and L_{MMI} are determined to be 7.5 µm and 240 µm with excess loss of less than 0.2 dB.

Optical transmitter module

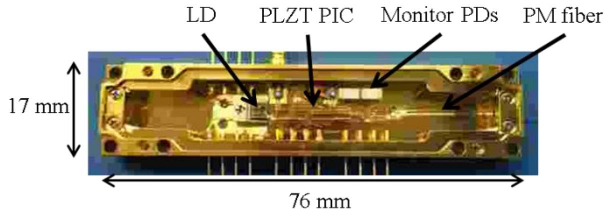

Figure S5 | Photograph of optical transmitter module. Monitor photodiodes (PDs), a laser diode (LD), and a polarization maintaining (PM) fiber are directly coupled to the PIC, which provided a low-optical-loss implementation of the transmitter module.
